# Coma aberrations in combined two- and three-dimensional STED nanoscopy

**DOI:** 10.1364/OL.41.003631

**Published:** 2016-08-01

**Authors:** Jacopo Antonello, Emil B. Kromann, Daniel Burke, Joerg Bewersdorf, Martin J. Booth

**Affiliations:** 1 Centre for Neural Circuits and Behaviour, University of Oxford, Mansfield Road, Oxford OX1 3SR, UK; 2 Department of Engineering Science, University of Oxford, Parks Road, Oxford OX1 3PJ, UK; 3 Department of Cell Biology, Yale School of Medicine, New Haven, Connecticut 06520, USA; 4 Department of Biomedical Engineering, Yale University, New Haven, Connecticut 06510, USA

**Keywords:** (180.6900) Three-dimensional microscopy, (100.6640) Superresolution, (050.1960) Diffraction theory, (180.2520) Fluorescence microscopy

## Abstract

Stimulated emission depletion (STED) microscopes, like all super-resolution methods, are sensitive to aberrations. Of particular importance are aberrations that affect the quality of the depletion focus, which requires a point of near-zero intensity surrounded by strong illumination. We present analysis, modeling, and experimental measurements that show the effects of coma aberrations on depletion patterns of two-dimensional (2D) and three-dimensional (3D) STED configurations. Specifically, we find that identical coma aberrations create focal shifts in opposite directions in 2D and 3D STED. This phenomenon could affect the precision of microscopic measurements and has ramifications for the efficacy of combined 2D/3D STED systems.

Super-resolution fluorescence microscopes provide image representations with resolutions measured in tens of nanometers or lower [1]. Stimulated emission depletion (STED) microscopy [2] obtains this resolution by restricting emitted fluorescence to a region much smaller than the diffraction limited excitation focus. This is achieved through the use of a depletion focus, which consists of a point of low (ideally zero) intensity surrounded by a region of higher intensity. Excited molecules in the high-intensity region are depleted through saturated stimulated emission to ensure that spontaneous fluorescence emission can only take place near the intensity minimum. The performance of these microscopes is critically dependent upon the quality of the depletion focus and the contrast between the minimum intensity point and the surrounding illumination. It has been shown [3] that aberrations can strongly affect the performance of these microscopes and adaptive optics has been introduced in order to overcome these problems [4–6].

The STED microscope consists of three beam paths: the excitation path, the emission path, and the depletion path. The excitation and emission paths together form, in effect, a confocal laser scanning microscope; the depletion path is co-linear with the excitation path and contains a phase mask that generates a ring-shaped depletion focus. The most common STED configurations provide either two-dimensional (2D) or three-dimensional (3D) resolution enhancement by the use of different phase masks, which can be implemented using a spatial light modulator (SLM) [7]. As all three paths pass through the same parts of the specimen on the way to the focus, they all suffer from the same aberrations. Although the beams may be at different wavelengths, they all experience the same optical path length aberration provided the media are nondispersive. Aberrations are introduced by spatial variations of the refractive index within the specimen or alignment errors in the optical system; specifically, coma can arise from miscentered lenses, a tilted coverslip, or common specimen structures [8,9]. The key to effective STED operation is the maintenance of a good zero at the center of the depletion focus. This zero is affected by aberrations. The 3D STED configuration is particularly sensitive [4] to such distortions. Previously [5], it was shown how misalignment of the phase mask can be mistakenly attributed to coma aberrations for the 2D or 3D STED modes. In this Letter we show that, to first approximation, small amounts of coma do not fill in the zero of the depletion focus but shift its position laterally by amounts that can be much larger than the resolution of the microscope. Moreover, we show that the shifts are in opposite directions for the 2D and 3D STED modes, even when using Zernike coma aberrations, which are defined to give zero shift in the scalar focusing approximation. This behavior has ramifications for the precision of measurements, particularly when switching among confocal, 2D, and 3D STED modes. It also implies that microscopes that combine the 2D and 3D depletion foci [10,11] are particularly susceptible to aberrations.

We model the electric field **E** of a depletion focus at the focal plane of a high numerical aperture (NA) objective using the Debye–Wolf integral [12,13]:

(1) $$\text{E}\left(x,y,z\right)=\int_{0}^{\alpha }\int_{0}^{2\pi }\text{e}\left(\theta ,\phi \right)T\left(\theta ,\phi \right)\exp\left[i\Phi \left(\theta ,\phi \right)\right]\times \exp\left[ik\left(x\sin\theta \cos\phi +y\sin\theta \sin\phi \right)\right]\times \exp\left[ikz\cos\theta \right]\sin\theta \;\text{d}\phi \;\text{d}\theta ,$$

where (*θ*, *ϕ*) and (*x*, *y*, *z*) are, respectively, the coordinates on the Gaussian reference sphere and in the focal region, as defined in [13]. *α* is the semi-aperture angle such that NA = *n* sin *α*, where *n* is the index of refraction, and *k* = *n*2*π*/*λ*, where *λ* is the wavelength. Note that in Eq. (1), we neglect some proportionality constants to maintain a simple notation, as these do not affect our results. For circularly polarized light, as commonly used in STED [14,15], the field distribution **e**(*θ*, *ϕ*) is

(2) $$\text{e}\left(\theta ,\phi \right)=A\left(\theta \right)\sqrt{\cos\theta }\left[\begin{array}{c} \cos\theta +1+\left(\cos\theta -1\right)e^{i2\phi }\\ i\left(\cos\theta +1\right)-i\left(\cos\theta -1\right)e^{i2\phi }\\ -2\sin\theta e^{i\phi } \end{array}\right],$$

where *A*(*θ*) accounts for the nonuniform illumination profile of the depletion beam, which is assumed here to be Gaussian. Φ(*θ*, *ϕ*) is the aberration function, and *T*(*θ*, *ϕ*) denotes the phase mask function for the 2D or 3D depletion beams, respectively:

(3) $$T_{2}\left(\phi \right)=e^{i\phi }\;T_{3}\left(\theta \right)=\{\begin{array}{rr} -1 & \theta \leq \beta \\ 1 & \theta >\beta \end{array},$$

where *β* is chosen to ensure zero electric field at the center of the depletion focus. We restrict our analysis to coma aberrations along the *x* axis, which are separable functions in polar coordinates and have cosine dependence on the azimuthal angle *ϕ*, i.e.,

(4) Φ(θ,ϕ)=cf(θ)cosϕ,

where *f*(*θ*) is an arbitrary radial function, e.g., the radial function of the primary Zernike coma [16], and *c* is a coefficient defining the magnitude of the aberration.

To simplify the analysis, while retaining the relevant phenomena, we consider the variation of the field **E** along the *x* axis (*y* = 0 and *z* = 0). From Eq. (1), we have

(5) $$\text{E}\left(x\right)=\int_{0}^{\alpha }\int_{0}^{2\pi }\text{e}\left(\theta ,\phi \right)T\left(\theta ,\phi \right)\times \exp\left[i\left(cf\left(\theta \right)+kx\;\sin\theta \right)\cos\phi \right]\sin\theta \;\text{d}\phi \;\text{d}\theta .$$

Further, for sufficiently small values of *c* and *x*, we can approximate the exponential in Eq. (5) with its first order Taylor expansion, i.e.,

(6) exp[i(cf(θ)+kx sinθ)cosϕ]≈1+i(cf(θ)+kx sinθ)cosϕ.

Substituting Eq. (6) and *T*_2_ into Eq. (5), we have the following approximation for the electric field of the 2D depletion focus **E**_2_:

(7) $${\text{E}}_{2}\left(x\right)\approx \int_{0}^{\alpha }\int_{0}^{2\pi }\left[\begin{array}{c} \left(\cos\theta +1\right)e^{i\phi }+\left(\cos\theta -1\right)e^{i3\phi }\\ i\left(\cos\theta +1\right)e^{i\phi }-i\left(\cos\theta -1\right)e^{i3\phi }\\ -2\;\sin\theta e^{i2\phi } \end{array}\right]\times \left[1+i\left(cf\left(\theta \right)+kx\sin\theta \right)\cos\phi \right]\times A\left(\theta \right)\sqrt{\cos\theta }\sin\theta \;\text{d}\phi \;\text{d}\theta .$$

Equation (7) can be simplified by evaluating the integrals in d*ϕ* and by exploiting the orthogonality properties of the trigonometric functions, so that

(8) $$\begin{array}{l} {\text{E}}_{2}(x)\approx \int_{0}^{\alpha }\left[\begin{array}{l} i\pi (\cos\theta +1)(cf(\theta )+kx\;\sin\theta )\\ -\pi (\cos\theta +1)(cf(\theta )+kx\;\sin\theta )\\ 0 \end{array}\right]\times A(\theta )\sqrt{\cos\theta }\sin\theta \;\text{d}\theta . \end{array}$$

Therefore, the condition **E**_2_(*x*) ≈ **0** is satisfied for

(9) $$\int_{0}^{\alpha }(\cos\theta +1)(cf(\theta )+kx\;\sin\theta )A(\theta )\sqrt{\cos\theta }\sin\theta \;\text{d}\theta \approx 0,$$

which, upon computing the integrals in d*θ* and replacing *x* with Δ*x*_2_, results in a linear equation, i.e.,

(10) $$a_{2}c+b_{2}k\Delta x_{2}\approx 0,$$

where *a*_2_ and *b*_2_ are the following constants:

(11) $$\begin{array}{l} a_{2}=\int_{0}^{\alpha }(\cos\theta +1)f(\theta )A(\theta )\sqrt{\cos\theta }\sin\theta \;\text{d}\theta \\ b_{2}=\int_{0}^{\alpha }(\cos\theta +1)A(\theta )\sqrt{\cos\theta }{\left(\sin\theta \right)}^{2}\;\text{d}\theta . \end{array}$$

Equation (10) shows that, within the validity range of the approximation in Eq. (6), the zero of the 2D depletion focus is translated along the *x* axis by a distance Δ*x*_2_, which is proportional to the amplitude *c* of the coma aberration defined in Eq. (4).

The same argument outlined in the preceding section can be repeated here for the 3D depletion focus. In this case, the electric field **E**_3_ is

(12) $$\begin{array}{l} {\text{E}}_{3}(x)\approx \int_{0}^{\alpha }\int_{0}^{2\pi }\left[\begin{array}{l} (\cos\theta +1)+(\cos\theta -1)e^{i2\phi }\\ i(\cos\theta +1)-i(\cos\theta -1)e^{i2\phi }\\ -2\;\sin\theta e^{i\phi } \end{array}\right]\times [1+i(cf(\theta )+kx\;\sin\theta )\;\cos\phi ]\times T_{3}(\theta )A(\theta )\sqrt{\cos\theta }\sin\theta \;\text{d}\phi \;\text{d}\theta . \end{array}$$

By computing the integrals in d*ϕ*, we have

(13) $${\text{E}}_{3}(x)\approx \int_{0}^{\alpha }\begin{array}{l} \left[\begin{array}{l} 2\pi (\cos\theta +1)\\ i2\pi (\cos\theta +1)\\ -i2\pi \;\sin\theta (cf(\theta )+kx\;\sin\theta ) \end{array}\right]\\ \times T_{3}(\theta )A(\theta )\sqrt{\cos\theta }\sin\theta \;\text{d}\theta . \end{array}$$

We assume that *T*_3_ is an optimal 3D phase mask, so that **E**_3_ is zero at the origin when *c* = 0. This condition is verified by choosing *β* such that the first two elements of the vector on the right-hand side of Eq. (13) vanish after integration, which can be performed analytically when *A*(*θ*) ≈ 1.

The requirement **E**_3_ ≈ **0** can then be reduced to a condition concerning only the last element of the vector, i.e.,

(14) $$\int_{0}^{\alpha }(cf(\theta )+kx\;\sin\theta )T_{3}(\theta )A(\theta )\sqrt{\cos\theta }{\left(\sin\theta \right)}^{2}\;\text{d}\theta \approx 0.$$

By computing the integrals in d*θ*, and replacing *x* with Δ*x*_3_, we derive the following linear equation:

(15) $$a_{3}c+b_{3}k\Delta x_{3}\approx 0.$$

The constants *a*_3_ and *b*_3_ are

(16) $$\begin{array}{l} a_{3}=\int_{0}^{\alpha }f(\theta )T_{3}(\theta )A(\theta )\sqrt{\cos\theta }{\left(\sin\theta \right)}^{2}\;\text{d}\theta \\ b_{3}=\int_{0}^{\alpha }T_{3}(\theta )A(\theta )\sqrt{\cos\theta }{\left(\sin\theta \right)}^{3}\;\text{d}\theta . \end{array}$$

This shows that small amplitudes of coma aberrations cause the zero intensity point of a 3D depletion focus to be maintained but also to translate along the axis.

In our STED microscope, which is described in [17], the 2D and 3D depletion beams are provided by a single laser source and a spatial light modulator, which are arranged as described in [11,18]. To simulate specimen-induced coma, we apply an aberration by adding it to the phase profile impressed on the SLM. For illustration purposes, we chose the coma aberration described by the Zernike polynomial [16] with radial order three and azimuthal order one, so that Eq. (4) becomes

(17) $$\Phi (r,\phi )=c\sqrt{8}(3r^{3}-2r)\;\cos\phi ,$$

where *r* is the radius in the unit disk of the pupil plane, which is related to the polar angle *θ* by *r* = *n* sin*θ*/NA, and *c* is the amount of coma in radians. The constants for Eq. (1) are *λ* = 775 nm, NA = 1.4, *n* = 1.5, and *β* = 0.62. By numerically integrating Eqs. (11) and (16), we obtained *a*_2_ = −0.3671, *b*_2_ = 0.3049, *a*_3_ = 0.1441, and *b*_3_ = 0.0722, which implies that the zeros move in opposite directions along the *x* axis. We report our measurements of the point-spread functions (PSF) of the 2D and 3D depletion foci, respectively, in Figs. 1(a) and 1(b), where we have subtracted the background counts. It can be seen that the zeros move in opposite directions as a function of the coma aberration *c*. As previously observed [4,19], we find that the 3D depletion PSF is more sensitive to aberrations; when |*c*| = 0.6 rad, one can still discern an isolated zero in the 2D PSF, whereas the ring of higher intensity is incomplete in the 3D PSF.

We implemented a heuristic function in MATLAB as a quantitative criterion to locate the zeros in the measurements of the PSF. This function selects the zero as the minimum pixel value within a closed level set that is nearest to the centroid of 20% of the brightest pixels. The shifts estimated using this function are reported using solid lines in Fig. 1(c). Note that, for clarity, we have subtracted an offset from each of these lines. These two offsets are due to residual misalignment, as can be seen by examining the PSFs in Figs. 1(a) and 1(b) when *c* = 0. The heuristic function successfully recovers the location of the zeros up to |*c*| ≤ 0.6 for the 2D case and up to |*c*| ≤ 0.4 for the 3D case. The predictions of the shifts obtained using Eqs. (10) and (15) are also reported within Fig. 1(c) using dashed lines. The results show the same trends in the shift of the zero position between the experimental measurements and modeling in both the 2D and 3D STED cases. The small mismatches between theory and measurement could be attributed to experimental conditions and the range of validity of the approximations used in Eq. (6).

Certain implementations [10,11] of STED microscopy use two incoherent beams to combine the 2D and 3D depletion foci and achieve superior lateral and axial resolution. The effectiveness of this method relies upon the precise co-alignment of the zero-intensity points of the two foci. It is clear from the results presented above that the presence of coma will cause a lateral shift that separates the zeros of the two foci, thus rendering a nonzero intensity at the center of the depletion pattern. This would occur even if a zero intensity were maintained in each of the foci separately, as illustrated in Fig. 2, where the normalized intensity cross sections are shown for the 2D (*I*_2_), 3D (*I*_3_), and combined (*I*_c_) depletion foci. The effect of coma on the contrast between the minimum intensity point and the surrounding illumination is quantified in Fig. 3, which reports the ratio between the minimum *I*_min_ and the maximum *I*_max_ of the cross section *I*_*c*_ as a function of the coma aberration *c*.

The disruption to the combined depletion foci can be seen by comparing the STED images within Figs. 4(c) and 4(d) with that within Fig. 4(b), which is not affected by coma. In these examples, coma aberrations of magnitude 0.8 lead to image shifts and significantly lower signal levels. The maximum intensity of the fluorescence drops by almost a factor of 10. These measurements were obtained by simultaneously applying the same amount of coma to the 2D and 3D depletion beams using the SLM. In this case, the two depletion beams were combined incoherently using orthogonal polarization states reflected twice on the SLM, as described in [11,18]. Note that the disruption of the depletion efficacy is not only due to the coma aberration itself but is also further exacerbated by the fact that the two zeros move apart. This can be seen by considering Figs. 4(e) and 4(f), where the same magnitude of coma aberration is applied with opposite signs to each depletion beam. In this latter case, the foci are equivalently deformed by the coma aberrations but move in the same direction. This results in an overall more efficient STED effect and in a less severe reduction of the signal than in Figs. 4(c) and 4(d).

We have shown that coma aberrations cause the zeros of the 2D and 3D STED foci to shift in opposite directions. Due to this phenomenon, we expect that microscopes that implement the combined 2D/3D STED mode are particularly susceptible to aberrations, indicating that aberration correction is likely to be essential in the wider application of these methods.

## Figures and Tables

**Fig. 1:**
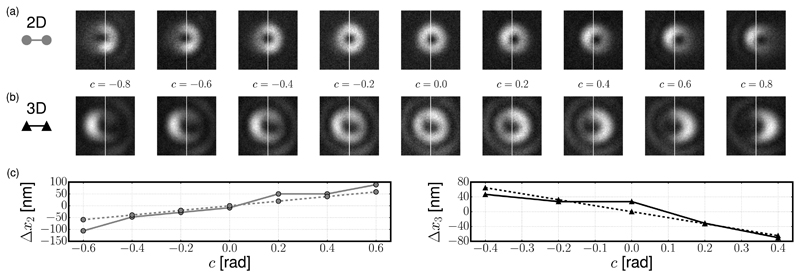
(a) *xy* cross sections of the 2D depletion focus measured by scanning a 150 nm gold bead for different values of the coma aberration *c*. The white vertical bars mark the center of each image (1.55 μm × 1.55 μm). (b) *xy* cross sections of the 3D depletion focus. Each image uses a different color map to enhance the contrast. (c) Shifts Δ*x*_2_ (left) and Δ*x*_3_ (right). Solid lines: shifts estimated from the data in (a) and (b). Dashed lines: shifts calculated using Eqs. (10) and (15).

**Fig. 2:**
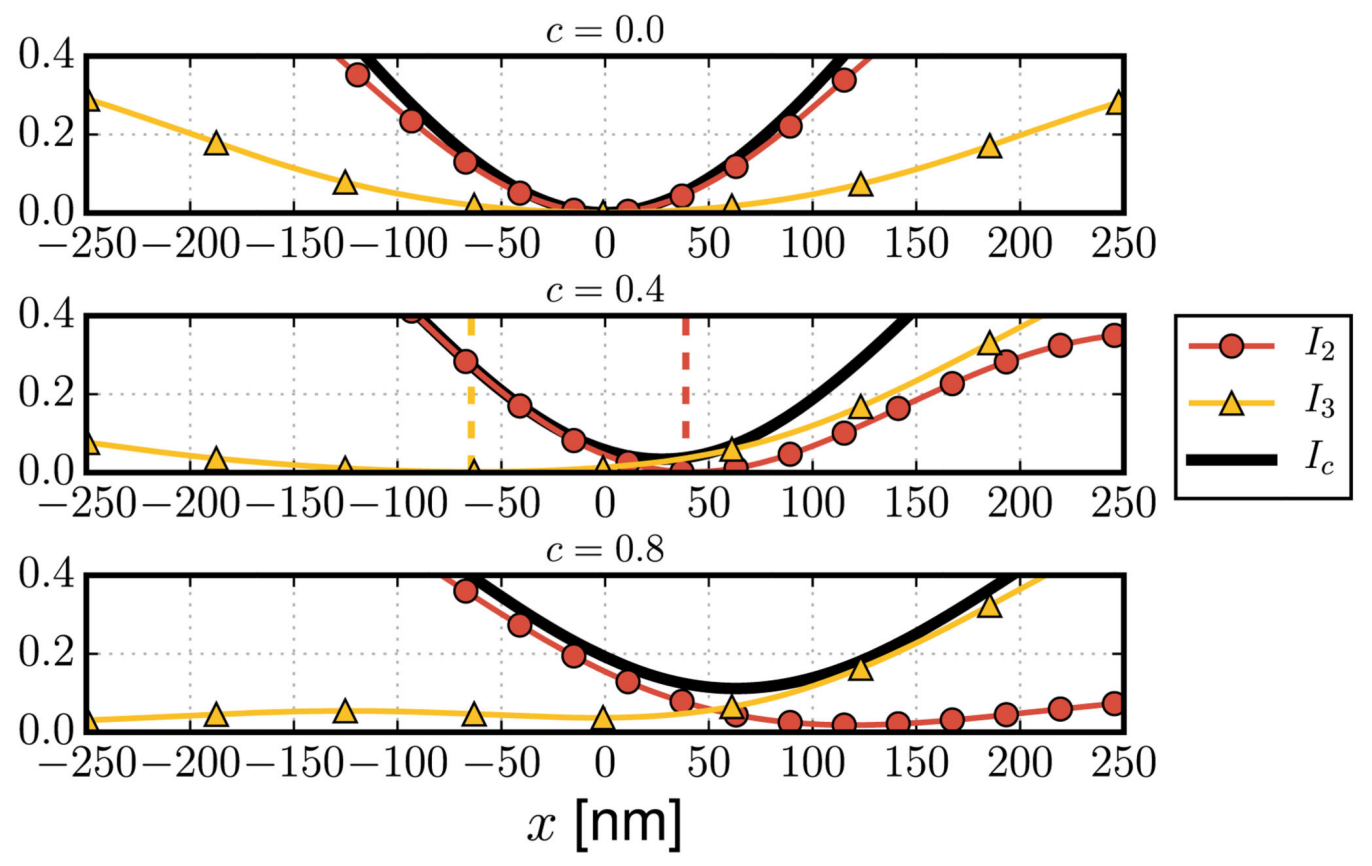
Effect of coma aberration *c* on the normalized intensity cross sections of the 2D (*I*_2_), 3D (*I*_3_), and combined (*I*_c_) depletion foci. Each curve, obtained by numerically evaluating the integrals in Eq. (1), is normalized to the maximum of *I*_c_ when *c* = 0. For *c* = 0.4, the vertical dashed bars denote the shifts Δ*x*_2_ = 39 nm and Δ*x*_3_ = −65 nm, according to Eqs. (10) and (15). The relative shift is 103 nm. For *c* = 0.8, *I*_3_ does not exhibit a well-defined zero.

**Fig. 3:**
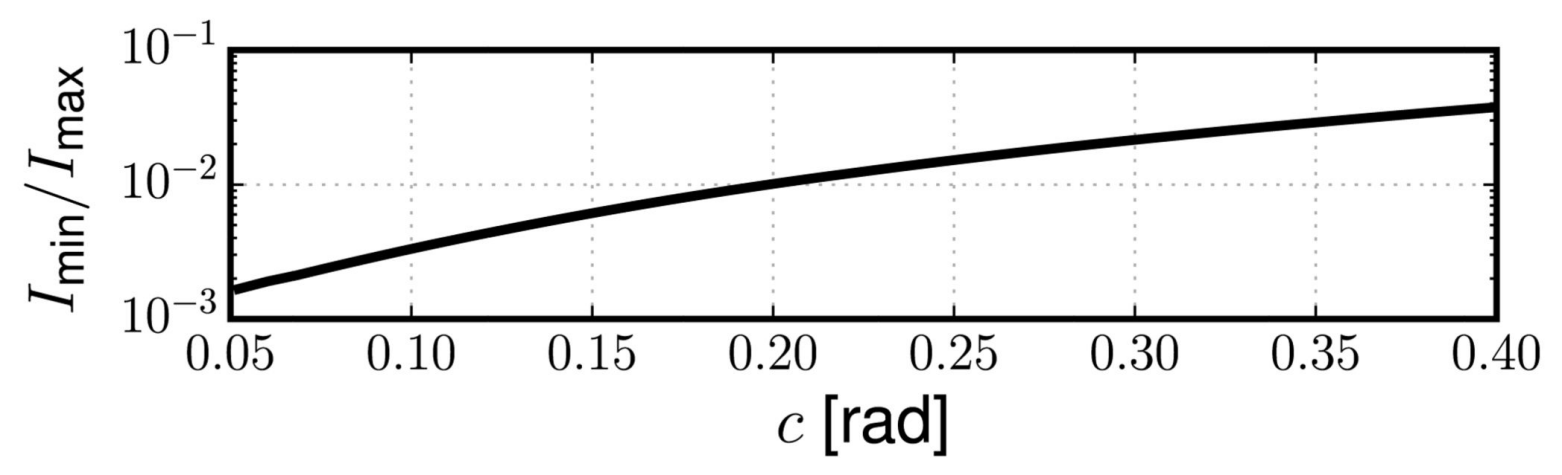
Ratio between the minimum (*I*_min_) and maximum (*I*_max_) of the intensity cross section (*I*_c_) of the combined depletion foci as a function of the coma aberration *c*. The graph is obtained by numerically evaluating the integrals in Eq. (1).

**Fig. 4:**
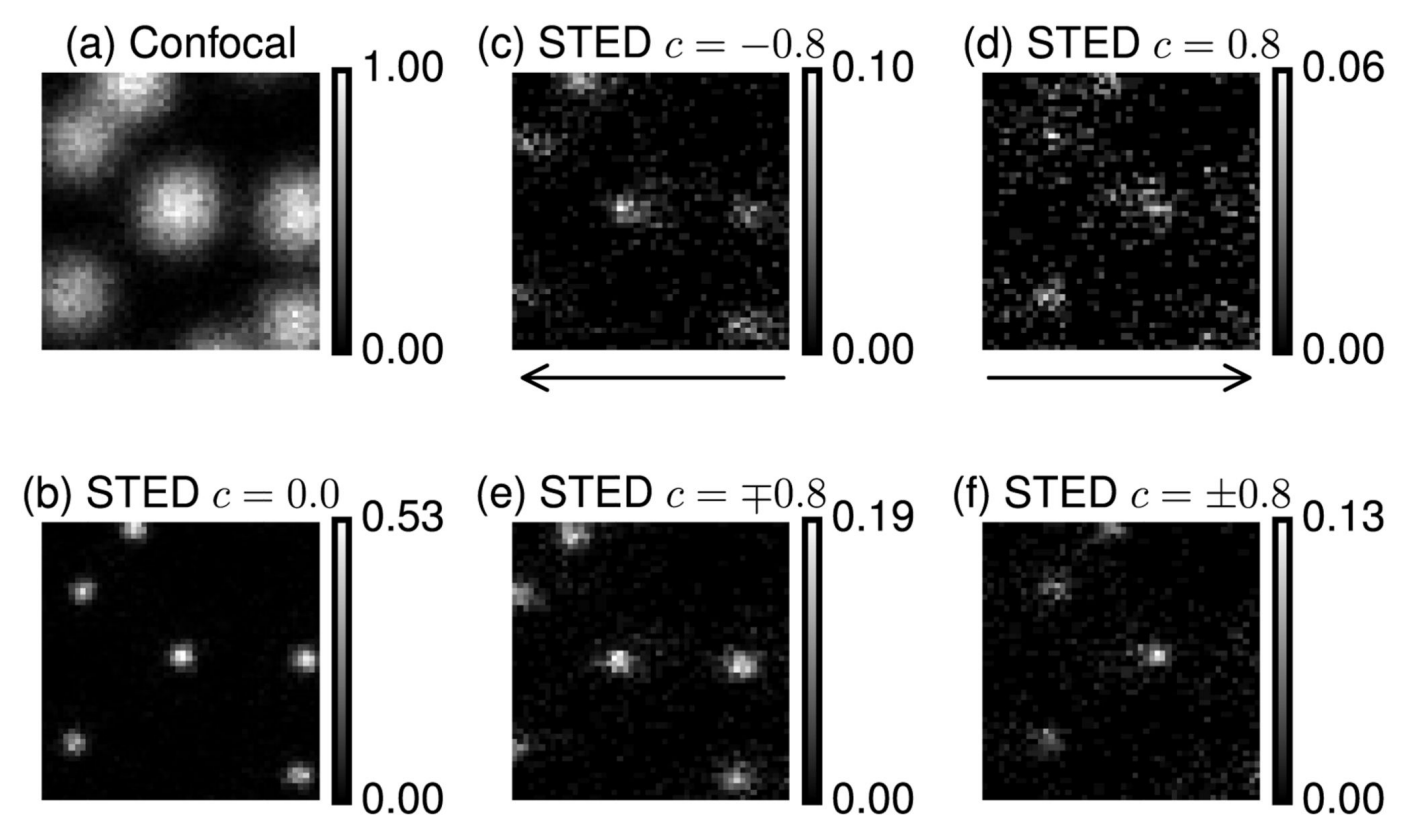
Images of 100 nm crimson beads. (a) Confocal mode. (b)–(f) Combined 2D/3D STED. (c) and (d) Same sign coma aberration applied to the depletion beams. Lateral shifts are indicated by the arrows, and significant reduction in signal is seen. (e) and (f) Opposite coma aberrations are applied to the depletion beams. As the zeros move in the same direction, the signal reduction is less pronounced. Image size: 1 μm × 1 μm.
